# Serum Extracellular Vesicle Stratifin Is a Biomarker of Perineural Invasion in Patients With Colorectal Cancer and Predicts Worse Prognosis

**DOI:** 10.3389/fonc.2022.912584

**Published:** 2022-07-22

**Authors:** Wenyun Hou, Meng Pan, Yi Xiao, Wei Ge

**Affiliations:** ^1^ Division of Colorectal Surgery, Department of General Surgery, Peking Union Medical College Hospital, Chinese Academy of Medical Sciences and Peking Union Medical College, Beijing, China; ^2^ National Key Laboratory of Medical Molecular Biology & Department of Immunology, Institute of Basic Medical Sciences, Chinese Academy of Medical Sciences, Beijing, China

**Keywords:** colorectal cancer, proteomics, extracellular vesicles, perineural invasion, epithelial-mesenchymal transition

## Abstract

Previous studies have shown that the presence of perineural invasion (PNI) is associated with a significantly worse prognosis in colorectal cancer (CRC) patients. In this study, we performed a detailed analysis of the diversity of extracellular vesicles (EV) between NPNI (non-PNI) and PNI using quantitative proteomics and aim to investigate the mechanisms underlying PNI in colorectal cancer. Quantitative proteomics technology was used to identify the proteome of serum-purified EVs from CRC patients with and without PNI (PNI and non-PNI (NPNI) groups, respectively) and healthy volunteers. Mass spectrometry data were verified by ELISA and Western blot analyses. The proteomic profile of serum EVs from the PNI group differed from that of those in the NPNI group. Serum-derived EVs from the PNI promoted more significant cellular mobility than EVs derived from the NPNI group. EV stratifin (SFN) expression levels demonstrated an area under the receiver operating characteristic curve values of 0.84 for discriminating patients with PNI from NPNI patients. Moreover, EV SFN expression levels were an independent predictor of CRC prognosis. In this study, we identified SFN as a potential biomarker for the diagnosis of PNI in stage II CRC patients.

## Introduction

Colorectal cancer (CRC) is the most frequently diagnosed cancer and the leading cause of cancer deaths worldwide. In the United States, it was estimated that 104,610 cases of colon cancer and 43,340 cases of rectal cancer would occur in 2020 (1). Perineural invasion (PNI) is a pathologic change characterized by tumor invasion of nervous structures and spread along nerve sheaths in the absence of lymphatic or vascular invasion (2). It has been proposed that the mechanisms of PNI involve complex signaling between tumor cells, stromal cells, and the nerves, although the precise details remain to be established (3–6). PNI is known to be a marker of aggressive disease and poor prognosis in several malignancies, including head-and-neck cancer (7), prostate cancer (8), and colorectal cancer (9). Several studies have demonstrated that the presence of PNI in CRC patients is related to a significantly worse prognosis (9–12). A retrospective analysis of 269 consecutive patients who had colorectal tumors resected revealed a four-fold greater 5-year survival rate in patients without PNI versus patients with PNI-positive tumors and strongly indicated the value of PNI as an independent prognostic factor in CRC (9). For stage II CRC patients, multivariate analysis revealed that patients with PNI showed a markedly worse 5-year disease-free survival (DFS) rate compared with those without PNI (13). In accordance with these results, a meta-analysis showed that PNI was associated with a poor outcome and the survival rate of stage II CRC patients with PNI was similar to that of patients with stage III disease (14). Another meta-analysis of 58 studies including 22,900 patients also showed that PNI is associated with worse 5-year overall survival (OS) and DFS (11). It has been demonstrated that stage II CRC patients with PNI who received adjuvant chemotherapy had improved survival compared to those who did not. However, the target genes and molecular mechanisms underlying PNI in stage II CRC require further clarification.

Extracellular vesicles (EVs) are membrane-bound tissue-derived organelles that contain a variety of molecular structures including RNA, protein, and metabolites (15), which mediate cell-cell communication through the transmission of these molecular messengers (16). EVs may also regulate metastasis by transferring molecules involved in epithelial-mesenchymal transition (17) or creating a permissive microenvironment for tumor metastases (18).

**Figure 1:**
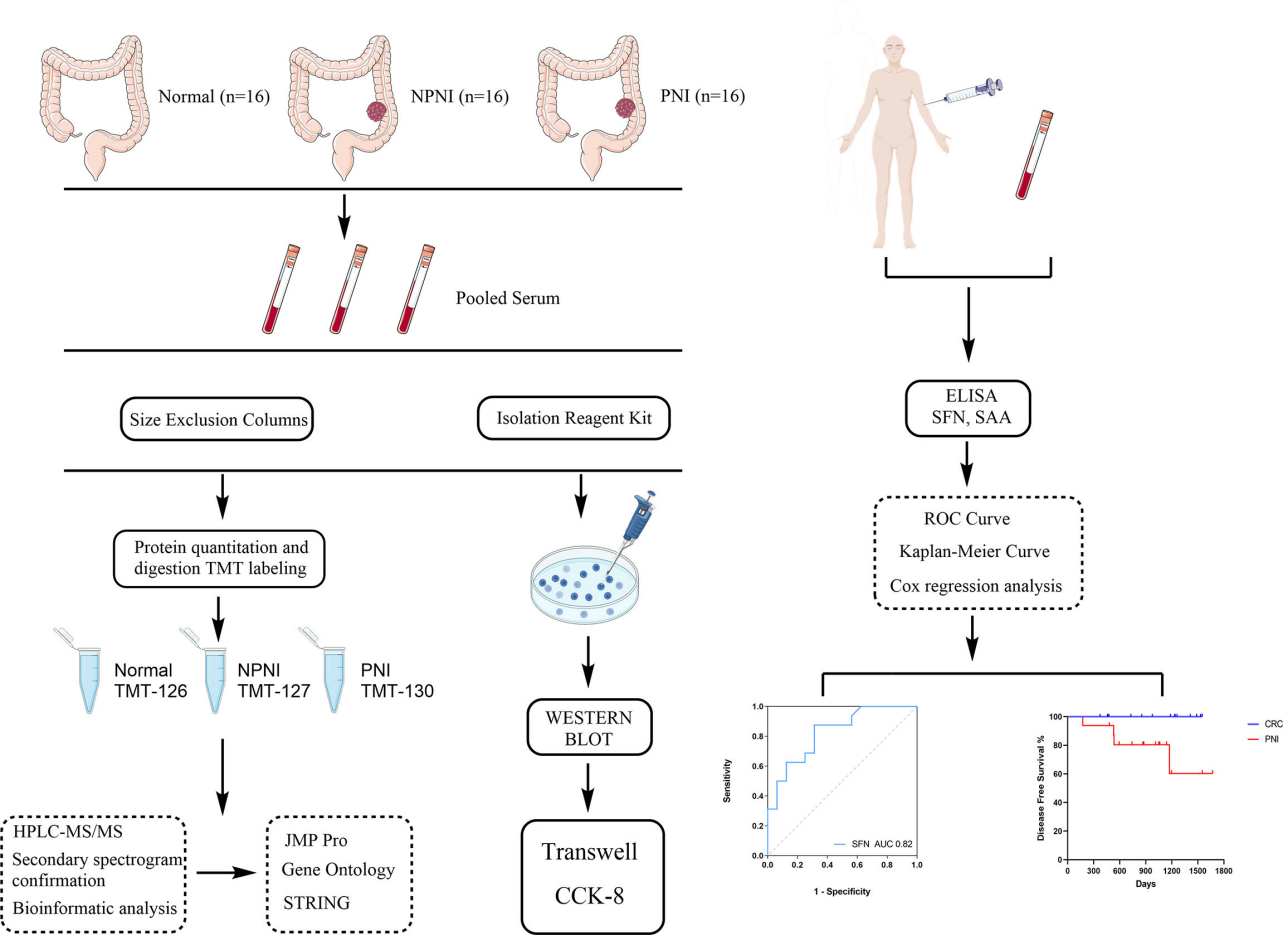
Schematic diagram of the experimental workflow employed in this study. The rectangular frame describes the analytical steps and tools employed in the proteomic study (see the “**Methods**” section for full details). PNI, perineural invasion; NPIN, non-perineural invasion; TMT, tandem mass tag; HPLC, high-performance liquid chromatography; mass spectrometry/mass spectrometry; DEP, differentially expressed protein; GO, gene ontology; ROC, receiver operating characteristic.

In this study, we used proteomic and bioinformatic strategies to determine the biological functions of EVs in CRC with PNI. Using tandem mass tag (TMT) label quantitation, we compared the proteome profiles of EVs purified from serum of stage II CRC patients with and without PNI (PNI and non-PNI (NPNI) groups, respectively) and healthy volunteers. Our study provides evidence that an understanding of the content of serum-purified EVs from patients might be useful for the evaluation of CRC with PNI and the development of novel diagnostics, therapeutics, and prognostic markers. A schematic diagram of the experimental workflow employed in this study is presented in **Figure 1**.

## Methods

### Patients and Healthy Controls

Patients with PNI, patients with NPNI, and healthy volunteers (n = 16 per group) were recruited at the Peking Union Medical College Hospital (PUMCH, Beijing, China) from 2017 to 2020. All enrolled patients met the following inclusion criteria: (a) stage II disease according to the TNM staging system; (b) diagnosed adenocarcinoma; (c) without lymph node and distant metastasis; (d) without a history of radiotherapy; (e) without diabetes; (f) without hematological diseases. The clinical information of CRC patients and healthy controls are shown in **Supplementary Table 1**. Postoperative surveillance and long-term follow-up were conducted according to the NCCN (national comprehensive cancer network) guidelines. Blood samples were collected before neoadjuvant therapy and radical surgery by percutaneous cubital venipuncture with a 21 G gauge needle. The whole blood was collected in a centrifuge tube and centrifuged at 3,000 ×*g* for 10 min to separate the serum, which was stored in new Eppendorf tubes at -80°C; each patient donated 3–5 mL serum. Serum samples from CRC patients with and without PNI and healthy volunteers (n = 16 per group) were pooled for further quantitative proteomics analysis; the pooled samples were allocated to the PNI, NPNI, and normal control groups. Informed consent was obtained from all patients. This study was approved by the Ethics Committee of Peking Union Medical College Hospital (No. S-k655) and conformed to the principles outlined in the Declaration of Helsinki.

### Isolation of EVs from Human Serum

Exosomes were isolated from the pooled serum samples of each group by size exclusion chromatography and the Total Exosome Isolation Reagent (Thermo Scientific) according to the manufacturer’s instructions. For size exclusion chromatography, qEV columns (Izon Science) were rinsed with phosphate-buffered saline (PBS) before adding the pooled serum samples (500 μl) and fractions (0.5 ml) were collected. Three EV-rich fractions (7–9) were pooled and concentrated using an Amicon Ultra-4 10 kDa centrifugal filter device (Merck Millipore). We also isolated EVs using the Total Exosome Isolation Reagent kit. The required volume of pooled serum sample was first diluted with an equal volume of PBS to decrease viscosity before adding 0.2 volumes of the Total Exosome Isolation Reagent. The mixture was vortexed and incubated at 4°C for 30 min. The sample was then centrifuged at 10,000 ×*g* for 10 min at room temperature and the pellet from every 100 μl serum was resuspended in 25 μl PBS for Western blotting and Transwell assays. A fraction of the resuspended isolated exosomes were lysed with RIPA buffer and the protein concentration was measured using a BCA protein assay kit (Thermo Scientific).

### Nanoparticle Tracking Analysis (NTA)

NTA was performed using a NanoSight LM10 instrument (Malvern Instruments Ltd). Aliquots from pooled EV-rich SEC fractions or exoEasy kit eluates were diluted in filtered PBS. Six videos of 30 s were captured for each sample.

### TMT Labeling and High-Performance Liquid Chromatography (HPLC) Analysis

EVs the healthy volunteer, NPNI, and PNI groups were labeled with TMT-126, TMT-127, and TMT-130, respectively, using the TMT labeling kit according to the manufacturer’s protocol (full details are presented in the supplemental methods). For HPLC analysis, the TMT-labeled peptides were loaded onto the Xbridge BEH300 C18 column controlled by the UltiMate 3000 HPLC workstation. A total of 4 to 7 fractions were collected at 1.5 min intervals. The 12 final fractions were combined for liquid chromatography-tandem mass spectrometry (LC)-MS/MS analysis.

### LC-MS/MS Analysis and Protein Identification

LC-MS/MS analysis of the peptides in the labeled digestion fractions was performed using an UltiMate 3000 System (Thermo Scientific) directly interfaced with the Thermo Orbitrap Fusion Lumos mass spectrometer (Thermo Scientific). The peptide samples were separated by gradient elution (120 min at 0.30 μl/min) across a fused silica capillary analytical column (75 μm internal diameter, 150 mm length; Upchurch, Oak Harbor, WA, USA) packed with C18 silica resin (300 Å, 5 μl; Varian, Lexington, MA, USA). The Orbitrap Fusion mass spectrometer was operated using Xcalibur 4.1 software in data-dependent acquisition mode with a single full-scan mass spectrum in Orbitrap (400–1,800 m/z, 60,000 resolution) followed by 3-s data-dependent MS/MS scans in an ion routing multipole at 35% normalized collision energy (HCD). The MS/MS spectra raw data were compared with the UniProt FASTA database (released on 5^th^ November 2021) using Proteome Discoverer 2.2 software (Thermo Scientific) following the software recommendations: full tryptic specificity required; two missed cleavages allowed; carbamidomethylation (C, +57.021 Da) and TMT-plex (K and peptide N-terminal) set as static modifications; dynamic modification, oxidation (methionine, M); mass tolerances for precursor ion and fragment ion set as 10 ppm (all MS acquired on an Orbitrap mass analyzer) and 20 mm (all MS2 spectra). The false discovery rate (FDR) for the identified proteins was set to 0.01(1%) and the reported ion intensities per peptide were used for relative protein quantification. Any protein with a fold-change >1.5 or <0.67 difference in expression between the groups was defined as a differentially expressed protein (DEP) (19–21).

### Bioinformation and Data Analysis

For proteomic analysis of serum-derived EVs, the relative abundance of proteins was presented as the ratios of TMT-127/126 (NPNI/normal group), TMT-130/126 (PNI/normal group), and 130/127 (PNI/NPNI group). The threshold of differential expression was set to >1.5-fold change. Both technical replicates were analyzed using Venn diagrams generated by Funrich software 3.1.3. Non-supervised principal component analysis (PCA) which was used to reduce the dimensionality of a data set consisting of a large number of interrelated variables and hierarchical cluster analysis of DEPs were performed using the JMP Pro 13.2.1 software (version 16.0.0, SAS Institute, Cary, NC, USA) and TBtools. Gene Ontology (GO) functional enrichment analysis and PCA were performed using DAVID 6.8 (https://david.ncifcrf.gov/) and the results were plotted using ggplot2 in the R program. Protein–protein interaction (PPI) networks describe physical interactions between proteins, taking place to mediate the assembly of proteins into protein complexes. Protein-protein interaction (PPI) analysis was performed and visualized using the string APP plugin in Cytoscape software (Version 3.7.2).

### Western Blot Analysis

Protein expression was evaluated by WB analysis. The protein concentration was determined using by BCA protein assay kit (Thermo Scientific). The proteins from lysed exosomes (15 μg per group) were then separated by sodium dodecyl sulfate polyacrylamide (10%) gel electrophoresis according to standard methods. The proteins were electrophoretically transferred to nitrocellulose filter membrane and blocked for 60 min with 5% non-fat dried milk in Tris-buffered saline plus 0.5% Tween. The membranes were then incubated overnight at 4°C with primary antibodies for the detection of the following: human ALIX (ab186429, Abcam; 1:1,000), human ALB (ab10241, Abcam; 1:1,000) and CD63 (sc-15363, Santa Cruz; 1:200), N-cadherin (#13116, CST; 1:1,000), GSK3β (ab68476, Abcam; 1:1,000), Slug (#9585, CST; 1:1000), MMP9(ab137867, Abcam; 1:1000), NF-κB (ab76302, Abcam; 1:1,000), and β-actin (GTX124213, GeneTex; 1:100000). Subsequently, the membranes were washed and incubated with horseradish peroxidase (HRP)-conjugated secondary antibodies (diluted at 1:10,000) and protein bands were visualized using electrochemiluminescent (ECL) reagents (Millipore, Bedford, MA).

### Cell Culture

SW480 and HCT116 cells were purchased from China Infrastructure of Cell Line Resources (Beijing, China). SW480 cells were maintained in Dulbecco’s Modified Eagle’s Medium (DMEM) containing 10% fetal bovine serum (FBS). HCT116 cells were cultured in Iscove’s modified Dulbecco’s medium (IMDM) containing 10% FBS. All cells were cultured at 37°C under 5% CO_2_ in a humidified atmosphere. The medium was changed every other day. Cells were passaged approximately every 2 days.

### Cell Proliferation Assay

SW480 and HCT116 cells were seeded in 96-well plates (3,000 cells/well). The relative viability of cells was evaluated by Cell Counting Kit-8 (CK04, Dojindo Laboratories) according to the manufacturer’s instructions. Absorbance was measured at 450 nm using a multi-microplate test system (Thermo Scientific).

### Cell Invasion and Migration Assays

Cell invasion assays were performed using Transwell Permeable Support 8.0 µm Polyester (PET) Membrane (6.5 mm) inserts coated with 10 µg diluted Matrigel Basement Membrane Matrix. SW480 or HCT116 cells were seeded into the upper chamber (3×10^4^ and 5×10^4^ cells per insert, respectively) in a 100 µl serum-free medium. Complete medium supplemented with 10% FBS was added outside the insert as a chemoattractant. Briefly, the EVs isolated from the serum of CRC patients or healthy volunteers were added to the top of the Transwell insert; an equal volume of exosome-free PBS was added as the blank control. The chamber was cultured for 24 h at 37°C under 5% CO_2_. The cells were then removed from the upper chamber and the chambers were fixed with methanol for 30 min. Cells outside the insert membrane were stained with 0.2% crystal violet for 60 min. The number of cells on the lower surface of the membrane was evaluated in three different fields (100× magnification). Three independent experiments were performed with triplicate wells. The protocol used for the migration assay was the same as that used for the invasion assay, except that the Transwell insert was not coated with Matrigel.

### ELISAs

For quantification of selected proteins, 100 μl serum-derived EVs were resuspended in 30 μl RIPA and diluted to 120 μl with sample diluent from the ELISA kit (Cusabio Biotech). The procedure was performed following the manufacturer’s instructions.

### Statistical Analysis

SPSS 26.0 (IBM Corp, Armonk, NY), JMP Pro, and GraphPad Prism (version 9.0; Nashville, TN, USA) software was used for all statistical analyses and data presentation. Variables with normal distribution were expressed as mean ± standard deviation (SD), while variables with non-normal distribution were expressed as the median and interquartile range unless otherwise indicated. Receiver operating characteristic (ROC) analysis was used to evaluate the performance of diagnostic tests and biomarkers and the area under the ROC curve (AUC) was calculated for each protein. The Cox proportional hazard ratio and 95% confidence interval information were also included. For all statistical methods used, *P* < 0.05 was set as the threshold for statistical significance.

## Results

### Project Design and Flow Chart of the Study

To explore the molecular mechanisms of PNI in CRC, we compared the differences in protein expression in serum EVs in the NPNI and PNI groups using TMT-based quantitative MS experiments. A flow chart of the study design is shown in **Figure 1**. The clinical information of the patients were presented in **Table 1**. The serum EVs of 32 CRC patients (16 with and 16 without NPI) and 16 healthy controls were isolated by size exclusion chromatography and the Total Exosome Isolation Reagent. The EVs isolated by size exclusion chromatography was used for TMT-based quantitative proteomics and those isolated using the specific reagent were used for WB and stimulation of the CRC cell lines. Expression of the EV markers CD63 and ALIX were detected by Western blotting. The particle diameters and concentration of EVs were quantified by NTA. The EV proteins identified by MS were validated by ELISA. Furthermore, the function of serum-derived EVs from both NPNI and PNI patients was evaluated by the Transwell assay. Subsequently, analysis of the clinical survival data of the patients revealed that SFN is related to colon cancer diagnosis and disease progression.

**Table 1:** Baseline characteristics of the patients.

Variable	NPNI group (N = 16)	PNI group (N = 16)	P
Sex			0.639
Male	7	7	
Female	9	9	
Age	60.75 ± 13.10	59.25 ± 13.37	0.751
T stage			0.394
T3	14	11	
T4	2	5	
N stage			/
N0	16	16	
N1-2	0	0	
Tumor deposit			/
Yes	16	16	
No	0	0	
MMR			0.484
dMMR	0	2	
pMMR	16	14	

### Extraction Efficiency and Verification of EVs

**Figure 2:**
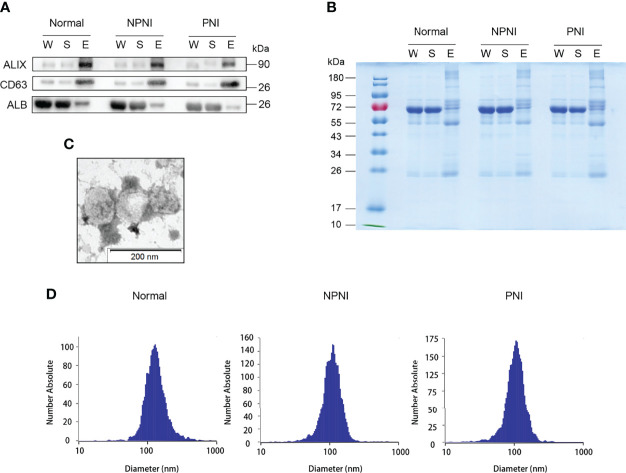
Extracellular vesicle (EV) purification and identification. **(A)** Purified EVs were verified by Western blot detection of EV markers ALIX and CD63. **(B)** Equal amounts of proteins from each sample were loaded. **(C)** Transmission electron micrographs of EVs; scale bar: 200 nm. Coomassie Blue staining of the total proteins separated by SDS-PAGE. **(D)** Nanoparticle tracking analysis (NTA) analysis of the EVs from the healthy control, NPNI, and PNI groups.

The EVs isolated using these two methods were used for WB and NTA analysis. WB analysis revealed that the EV surface markers CD63 and ALIX were both enriched in the EV fraction of all three groups, but not in whole serum and the supernatant fractions; the opposite pattern of enrichment was observed for the marker ALB (**Figures 2A, B**). The separated EVs were observed by TEM (**Figure 2C**). NTA showed that mean particle diameters of EVs in the healthy control, NPNI, and PNI groups were 126.8 nm, 111.6 nm, and 109.5 nm, respectively, with no significant differences between the three groups (**Figure 2D**). In addition, there were no significant differences in the particle concentration of EVs between healthy controls and CRC patients.

### Characteristics of DEPs in EVs Between the PNI and NPNI Groups

**Figure 3:**
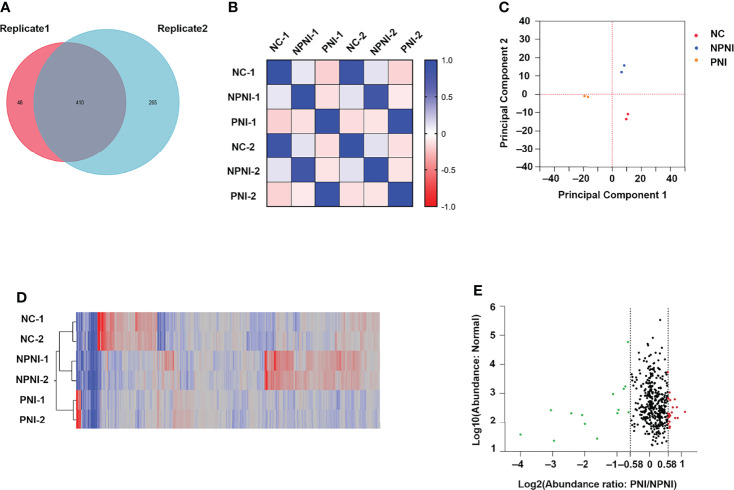
Characteristics of DEPs in EVs between the PNI and NPNI groups. **(A)** Venn diagram of proteins identified in duplicate quantitative TMT experiments, showing the overlap of 410 differentially expressed proteins (DEPs) identified in two technical replicates. **(B)** Correlation matrix showing the protein abundance correlations between the normal, NPNI, and PNI groups. **(C)** Principal component analysis of proteins identified in quantitative TMT experiments. **(D)** Heatmap showing the relationship between protein expression levels in each sample mapped using the 410 (DEPs) identified in two technical replicates of quantitative TMT experiments. **(E)** Scatter plot showing the distribution of upregulated (red dots) and downregulated (green dots) DEPs between the NPNI and PNI groups.

Quantitative TMT experiments were performed twice and a total of 456 proteins were identified in technical replicate 1 and 675 proteins (FDR <0.01, unique peptide ≥1) were identified in technical replicate 2. A total of 410 proteins were identified in both experiments (**Figure 3A**). Correlation analysis showed a high degree of correlation between the two replicate experiments (**Figure 3B**). Similar protein distributions were obtained in two technical replicates which were consistent with the results of the PCA (**Figure 3C**). The heatmap suggested a high degree of consistency in protein expression profiles detected in the two technical assays (**Figure 3D**). These results indicated a high degree of similarity between the protein profiles obtained in the two technical TMT-MS/MS assays. Next, we analyzed the average ratio of the 410 overlapping proteins. Based on a threshold of >1.5-fold change in expression of EV proteins, 37 DEPs were identified in the comparison of the PNI group versus the NPNI group consisting of 14 upregulated proteins (relative protein abundances >1.5) and 23 downregulated proteins (relative protein abundances <0.67) (**Figure 3E**). Full details are shown in **Supplementary Table 2**.

### GO Analysis of DEPs in EVs Between NPNI Group and PNI group

**Figure 4:**
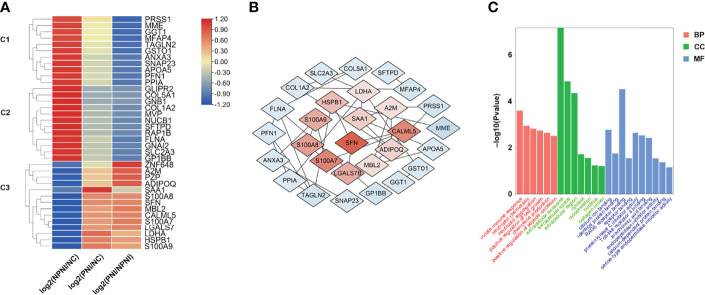
Hierarchical clustering analysis and heatmap of DEPs. **(A)** The heatmap was constructed based on a log2 transformation of relative abundance ratios (NPNI/PNI). **(B)** Protein-protein interaction (PPI) network analysis of differentially expressed proteins (DEPs) between the NPNI and PNI groups. **(C)** Gene ontology annotation of upregulated proteins enriched in Cluster 3 based on biological process, cellular component, and molecular function.

To provide insights into the biological significance of EV DEPs in the PNI group, the ratios of 37 DEPs were separated into three distinct groups (NPNI/Normal, PNI/normal, and PNI/NPNI) by hierarchical clustering using TBtools. Among the upregulated proteins, S100 family proteins, SFN, LDHA, and HSPB1 were all cancer-related proteins, which play important roles in tumor migration and invasion. (**Figure 4A**). PPI networks were visualized by Cytoscape based on their STRING fractions (**Figure 4B**). In Cluster 3, 14 DEPs were gradually upregulated in CRC patients with PNI, whereas inverse patterns were detected for 23 DEPs in Clusters 1 and 2 (**Supplementary Table 3**). In particular, we clustered the 14 upregulated DEPs into GO categories using DAVID online tool (**Figure 4C**). Molecular function analysis revealed that these proteins were mainly involved in calcium ion binding, identical protein binding, and RAGE binding. In addition, the DEPs related to biological process showed significant enrichment in the categories of positive regulation of cell growth. Cellular component analysis revealed that the DEPs were enriched mainly in the categories of extracellular exosome, extracellular space, and extracellular region, which indicated that the purification of EVs from serum was successful (**Supplementary Table 4**). The proteins SFN and SAA1 were clustered in the category of positive regulation of cell growth. The expression of these two proteins was then verified by ELISA. These results suggested that EVs of patients with PNI play an important role in cancer progression.

### Serum EVs From CRC Patients With PNI Promote Cellular Invasion and Migration

**Figure 5:**
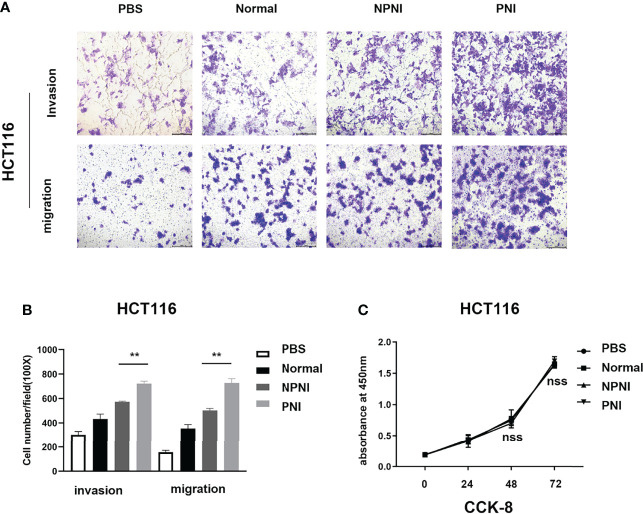
EVs derived from CRC patients with PNI promote CRC cell migration and invasion. **(A, B)** Transwell assays were used to determine the invasion and migration of HCT116 cells treated with EVs (175 μg) from CRC patients with PNI, CRC patients without PNI, or EVs from normal controls; PBS was used as a blank control. **(C)** Cell Counting Kit-8 assay of HCT116 cell proliferation following treatment with serum EVs derived from CRC patients with PNI, CRC patients without PNI, and normal controls. **P < 0.01, nss, *P* > 0.05 (Student’s *t*-test).

We performed Transwell assays to explore the effects of EVs (175 µg) on the invasion and migration properties of two colon cancer cell lines, HCT116 and SW480. EVs in the PNI group significantly increased the invasion and migration ability of HCT116 cells (**Figures 5A, B**). Additionally, EVs in the PNI group showed a significantly promotion in invasion and a tendency to promote tumor migration of SW480 cells. However, there is no significant statistical difference between the NPNI group and PNI group in migration for SW480 cells. (**Supplementary Figures 1A, B**). However, the proliferation of CRC cell lines was not promoted by EVs from either the NPNI or PNI groups (Student’s *t*-test, *P* > 0.05; **Figure 5C**). These results indicated that EVs from CRC patients with PNI showed a greater effect in promoting CRC invasion and metastasis compared with the effects of EVs from CRC patients without PNI.

### Evs Derived from CRC Patients With PNI Correlate With EMT and Promote Cancer Development *In Vitro*

**Figure 6:**
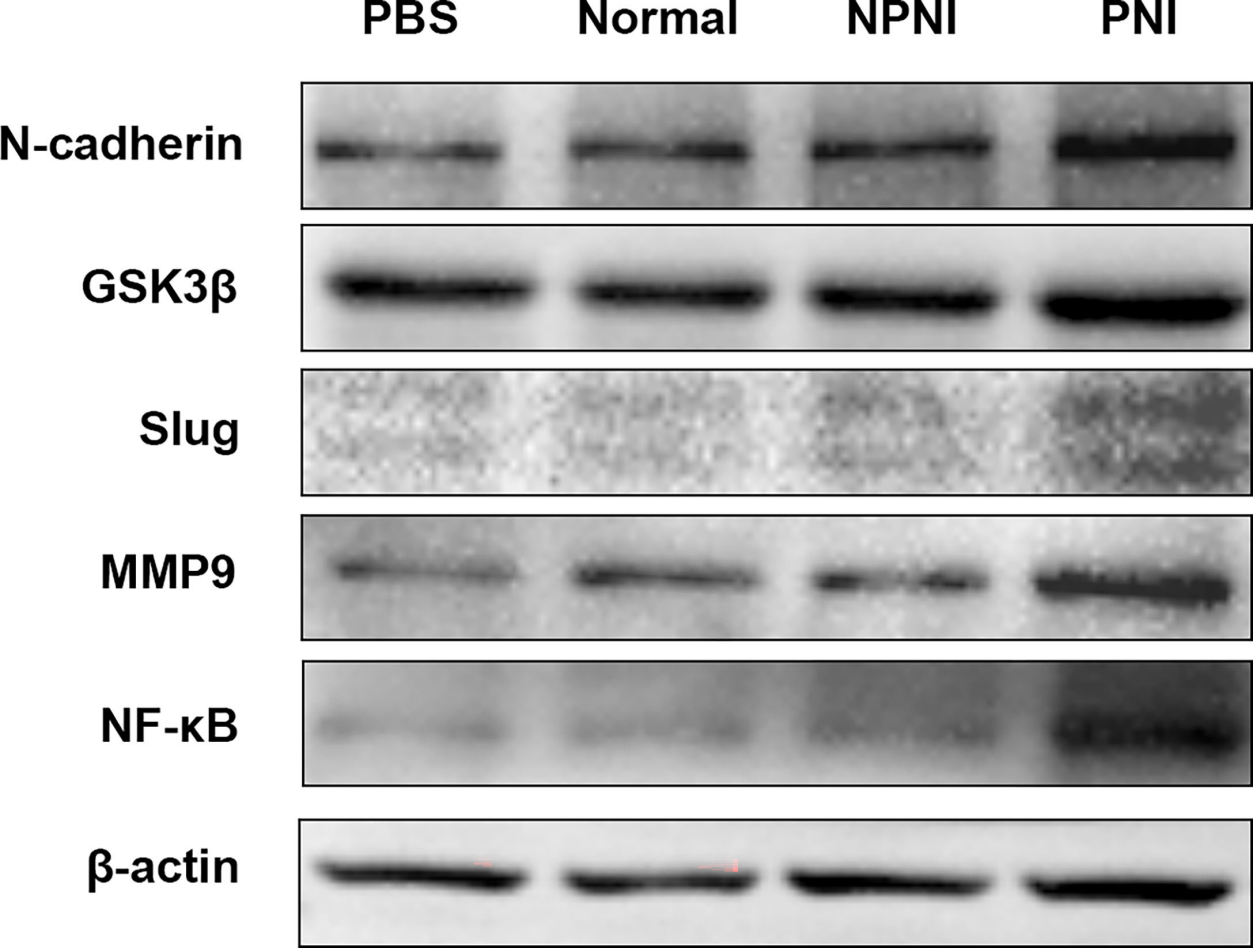
EMT-related protein expression in SW480 cells stimulated with EVs. Western blot analysis of N-cadherin, GSK-3β, Slug, MMP9, and NF-kB protein expression in SW480 cells stimulated with PBS and serum-derived EVs from the healthy volunteer, PNI and NPNI groups.

In further studies, we evaluated the expression of EMT-related proteins in SW480 cells stimulated for 24 h with serum-derived EVs. WB analysis revealed that the expression of N-cadherin, Slug, GSK-3β, NF-κB, and MMP9 was upregulated by EVs from the PNI group. (**Figure 6**). Therefore, we speculated that EVs derived from CRC patients with PNI may upregulate the expression of EMT-related proteins to promote tumor progression.

### Verification of Protein Expression Levels by ELISA

**Figure 7:**
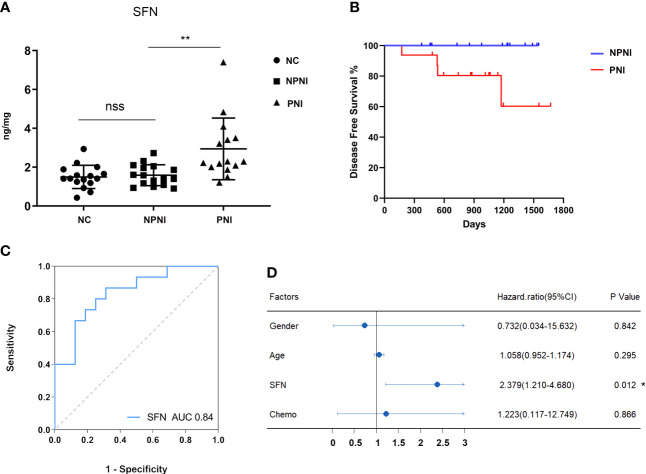
Verification of protein expression, and translational relevance of EV SFN. **(A)** Serum EVs from the NPNI (n = 16), PNI (n = 16) and healthy volunteer (n = 16) groups were analyzed by ELISA using antibodies for the specific detection of SFN. nss, P > 0.05, **P < 0.001 (Student’s t-test). **(B)** Kaplan–Meier analysis of disease-free survival (DFS) in CRC patients with and without PNI. **(C)** The diagnostic performances of EV SFN (AUC = 0.84). **(D)** Cox proportional hazards model indicates that SFN (HR, 2.379; 95% CI, 1.210–4.680; P < 0.05) is an independent prognostic factor for DFS. *P < 0.05.

Our bioinformatic analysis indicated that the DEPs in serum-derived EVs from CRC patients with PNI were enriched in positive regulation of cell growth. Both SFN and SAA1 were significantly more abundant in the PNI group than the NPNI group according to the MS/MS analyses. Therefore, we selected SFN and SAA1 as representative proteins for verification of these data by ELISA. Compared with the NPNI group, EV expression of SFN was found to be elevated in the PNI group (**Figure 7A**), while there was no significant difference in the EV expression of SAA1 between these two groups (**Supplementary Figure 2A**).

### SFN Is Related to PNI and Disease Progression in CRC Patients

Furthermore, Kaplan–Meier survival analysis of the 32 CRC patients indicated inferior DFS among patients with PNI compared with those without PNI (log-rank test, P < 0.05, **Figure 7B**). Moreover, ROC curve analysis revealed that EV SFN expression can be used to distinguish CRC patients with PNI from those without PNI, with an AUC of 0.84 (**Figure 7C**), whereas EV SAA1 expression showed no discrimination value (**Supplementary Figure 2B**). Furthermore, multivariate analysis (variables: sex, chemotherapy history, PNI status, serum levels of EV SFN) using the Cox proportional hazards model implicated SFN (HR: 2.379; 95% CI: 1.210–4.680; P < 0.05) as an independent prognostic factor for DFS (**Figure 7D**).

## DISCUSSION

Several studies have demonstrated that PNI is an independent prognostic factor for cancer-specific, overall, and disease-free survival in patients with CRC (9, 10). However, the molecular mechanisms underlying the poor prognosis of patients with PNI have not been clearly defined. In this study, we used high throughput TMT-labeled quantitative proteomics analysis to investigate the variations in EV protein expression between patients with and without PNI. We identified 37 DEPs involved in multiple processes related to cancer progression, including epithelial cell migration, tumor cell growth.

### EVs in CRC Patients With PNI Play Pivotal Roles in Cellular Invasion and Migration

Our Transwell experiments revealed that serum-derived EVs from CRC patients with PNI enhanced the migration and invasion ability of SW480 and HCT116 colon cancer cell lines. These results indicate that EVs derived from CRC patients with PNI contain certain proteins that promote metastasis. Among the upregulated proteins, we found that most were cancer-related proteins such as S100 family proteins, SFN, LDHA, and HSPB1, which all play important roles in tumor migration and invasion.

In this study, EVs derived from CRC patients with PNI were shown to be rich in signal transduction molecules such as S100A8, S100A7, and S100A9, with all three S100 family proteins found to be markedly upregulated. It has been reported that extracellular S100A8/A9 proteins secreted by myeloid-derived suppressor cells promote leukocyte recruitment, angiogenesis, tumor migration, and formation of pre-metastatic niches in distal metastatic organs through activation of the MAPK and NF-κB signaling pathways (22). A previous study indicated that pre-metastatic niche formation required S100 protein upregulation by lung resident cells (23). Silva and colleagues discovered that S100A8 or A9 regulated tumor cell migration and invasion by upregulating the expression of matrix metalloproteinases (24). Furthermore, Hoshino et al. demonstrated that exosome integrins taken up by resident cells activated proinflammatory S100 gene expression (25). Therefore, S100A8/A9 in EVs are implicated in the formation of premetastatic niches in distal metastatic organs and tumor cell migration in colon cancer (22). Additionally, several studies have demonstrated that 14-3-3 family members regulate actin dynamics by stabilizing cofilin phosphorylation or inhibiting signals *via* the AKT-RhoA pathway (26–28). In addition, Aaron et al. discovered that 14-3-3σ promoted cell migration and tumor invasion independently of proliferation by regulating cytoskeletal solubility and dynamics in breast tumors (29). In accordance with our proteomic data, ELISAs confirmed that the expression of SFN in serum EVs from CRC patients with PNI was markedly increased. All these previous studies indicated that SFN plays a critical role in the migration and invasion of cancer cells, although the mechanism underlying the function of SFN in CRC invasion and migration has not been elucidated. Lactate dehydrogenase A (LDHA) is an enzyme that plays a key role in regulating glycolysis. Several studies have suggested that aberrantly high expression of LDHA in numerous cancers significantly affects the invasion and migration of malignant cells (30). It has also been reported that LDHA phosphorylation and activation promote cancer cell invasion and metastasis (31). In particular, it was demonstrated that suppression of LDHA activity inhibited cell growth, suppressed tumor invasion, and induced apoptosis in GC cells (32). Koukourakis et al. identified a positive correlation between LDHA expression and distant metastasis of CRC (33). Furthermore, inhibition of LDHA activity resulted in reduced tumor growth in hereditary leiomyomatosis and renal cell carcinoma syndromes (34). Collectively, these findings indicate that proteins that are upregulated in serum-derived EVs of CRC patients with PNI promote tumor cell invasion and metastasis.

### EVs in CRC Patients With PNI Play Critical Roles in EMT Transition

EMT plays a key role in the early events of tumor metastasis in carcinoma cells (35). We initially found the changes in morphology of SW480 cells stimulation by EVs during the cell culture. Therefore, we speculated that SW480 cells may undergo the process of epithelial-mesenchymal transition. And we validated and analyzed the expression of EMT-related proteins in SW480 cell line stimulated with EVs. WB analysis showed that the expression of EMT-related proteins increased significantly in SW480 CRC cells stimulated with serum-derived EVs from CRC patients with PNI. To provide a glimpse into the role of serum EVs in EMT, we identified that the up-regulated DEPs were perhaps related to modulating the progression of EMT according to several studies. Several mediators of EMT were identified in our study, including S100 family proteins, LDHA and HSPB1. Studies have shown that S100A8 expression is gradually increased in normal stroma, tumor center stroma, and the tumor invasive front stroma, suggesting that S100A8 is involved in the regulation of EMT in CRC (36, 37). In particular, TGF-β activated the USF2/S100A8 signaling axis of CRC cells to promote EMT and metastasis in CRC (38). Moreover, extracellular S100A8/A9 can regulate breast cancer aggressiveness by inducing EMT *via* the MCAM/ETV4 axis (39). Therefore, we hypothesized that S100 family proteins in EVs promote EMT and metastasis in CRC, although this requires verification *in vitro* and *in vivo*. LDHA converts pyruvate and NADH to lactate (40), which provides an acidic microenvironment for tumor growth, while also contributing to the EMT process (41, 42). LDHA inhibitor reversed EMT-like phenomena while reducing the invasion and migration of tamoxifen-resistant breast cancer cells, thus confirming the function of LDHA in EMT regulation (43). HSPB1 is an ATP-independent molecular chaperone that is highly related to inflammatory cytokines, growth factors, and anticancer agents (44, 45). HSPB1 has been identified as an essential mediator in cancer progression and preventing apoptosis in transformed cells (46, 47). In particular, HSPB1 promoted migration and invasion in breast cancer cells (48), modulated EMT of lung cancer cells (49), and promoted EMT in prostate cancer (50). Collectively, our data showed that HSPB1 was aberrantly overexpressed in serum-derived EVs from CRC patients with PNI, thus implicating HSPB1 as a predictive marker of PNI in CRC. Based on these findings, we speculate that EVs of CRC patients with PNI promotes EMT and metastatic dissemination.

### SFN Is a Potential Biomarker for PNI Diagnosis and Prognosis in CRC Patients

SFN (14-3-3 sigma) is a cell cycle checkpoint and an adapter protein implicated in the regulation of a large spectrum of signaling pathways. SFN has been reported to play an important role in multiple kinds of tumors, including lung (51, 52), breast (53), ovary (54), bile duct (55), and gallbladder (56) cancers. SFN is encoded by a p53-inducible gene that is responsive to DNA damage (57), allowing SFN to function as a negative regulator of tumor cell cycle progression (58). However, accumulating evidence indicates that SFN also promotes tumor progression. A recent study demonstrated that SFN overexpression stimulated tumor initiation and progression of lung cancer (51). It was also shown that SFN regulates receptor tyrosine kinase (RTK) stabilization through its interaction with ubiquitin-specific protease 8 (USP8) (53). Kim et al. also found that SFN expression levels were significantly associated with pathological stage, lymphatic invasion, and vascular invasion. Moreover, SFN expression was associated with significantly worse outcomes relative to SFN-negative lung adenocarcinoma patients (P = 0.007) (52). High SFN expression is also associated with significantly worse OS for patients with ovarian serous adenocarcinoma who have received chemotherapy (54). In accordance with previous studies, SFN was aberrantly overexpressed in serum-derived EVs from CRC patients with NPI compared with those without NPI in our study. In addition, we identified SFN in EVs as an independent prognostic factor of DFS in CRC patients.

Although SFN plays important role in various cancers, its role in CRC is still obscure. To date, only a few studies have characterized the expression levels of SFN in CRC (57–59), although it has been reported that SFN mRNA expression is significantly decreased in CRC tissue (59). However, in our study, we discovered that SFN expression was obviously upregulated in serum-derived EVs of CRC patients with PNI compared to those without PNI. It can be speculated that this discrepancy is due to differences in the types of biological samples analyzed in these two studies. Furthermore, we identified serum SFN levels as a promising circulating biomarker that can be used to distinguish CRC patients with PNI from those without, with an AUC value of 0.84. However, this remains to be confirmed in a larger validation cohort.

### Higher Expression of SAA1 in EVs May Associate With Tumor Progression in CRC

Serum amyloid A (SAA) is a hallmark of the acute-phase response, which is associated with inflammatory conditions such as tissue injury, infection, and surgery (60). Human SAA1 is one of four SAA proteins and the best characterized in terms of its physiological functions. In the current study, we found that SAA1 expression was significantly upregulated in serum-derived EVs in the PNI group. Accumulating evidence indicates that increased SAA expression is related to tumor pathogenesis (61–63). For lung cancer patients undergoing EGF receptor tyrosine-kinase inhibitor therapy, increased plasma SAA1 expression is regarded as a biomarker of poor prognosis (64). In addition, a previous study demonstrated a close correlation between certain SAA1 allelic variants and nasopharyngeal carcinoma, some of which exhibited anti-angiogenic and tumor-suppressive activities (65). Stimulation of angiogenesis and matrix degradation (66) as well as upregulated expression of matrix-metalloproteinase-9 (67) may contribute to tumor metastasis and growth. It was recently reported that S100A4 enhanced the expression of mouse SAA1 and SAA3, which stimulated expression of RANTES, G-CSF, MMP2, S100A8, and S100A9 to promote tumor metastasis (68). Interestingly, our proteomic profiling studies revealed upregulation of S100 family proteins, such as S100A7, S100A8, and S100A9. However, our small-scale ELISA data showed that there were no significant differences in SAA1 levels between the PNI and NPNI groups and showed no diagnostic or prognostic value for CRC patients with PNI. This discrepancy may be due to the limited number of samples, thus, the potential of SAA1 as a prognostic marker for PNI in patients with CRC warrants further investigation.

## Conclusion

In summary, the expression profile of the serum EV proteome in patients with PNI differs from that of patients without PNI. Serum-derived EVs from CRC patients with PNI significantly promote CRC cell metastasis compared with those from patients without PNI. This difference may be due to the upregulation of the EMT process. But as a limitation of our study, the results of EVs from PNI group promoting tumor cell invasion and migration through EMT require confirmation by other CRC cell lines. Furthermore, EV SFN is implicated as a non-invasive diagnostic and prognostic biomarker of colon cancer with PNI.

## Data Availability Statement

The data presented in the study are deposited in the iProx repository, accession number PXD035096.

## Ethics Statement

The studies involving human participants were reviewed and approved by the ethics committee of Peking Union Medical College Hospital (No. S-k655). The patients/participants provided their written informed consent to participate in this study.

## Author Contributions

WG, YX, and WH: conceptualization, methodology, formal analysis, validation, and supervision. MP and WH: experimental studies, data curation, and writing-Original draft preparation. MP: statistical analysis and visualization. YX: funding acquisition. WG: writing–review and editing. All authors contributed to the article and approved the submitted version.

## Funding

This study was supported by grants from the Non-profit Central Research Institute Fund of the Chinese Academy of Medical Sciences (grant number 2019XK32003) and National Natural Science Foundation of China (grant number 62172437).

## Conflict of Interest

The authors declare that the research was conducted in the absence of any commercial or financial relationships that could be construed as a potential conflict of interest.

## Publisher’s Note

All claims expressed in this article are solely those of the authors and do not necessarily represent those of their affiliated organizations, or those of the publisher, the editors and the reviewers. Any product that may be evaluated in this article, or claim that may be made by its manufacturer, is not guaranteed or endorsed by the publisher.
