# Supplementary figures and images for: Serum Extracellular Vesicle Stratifin Is a Biomarker of Perineural Invasion in Patients With Colorectal Cancer and Predicts Worse Prognosis
Source: Front Oncol. 2022 Jul 22;12:912584. doi: 10.3389/fonc.2022.912584 (PMC9353013; doi:10.3389/fonc.2022.912584)

Figure 1

Alix
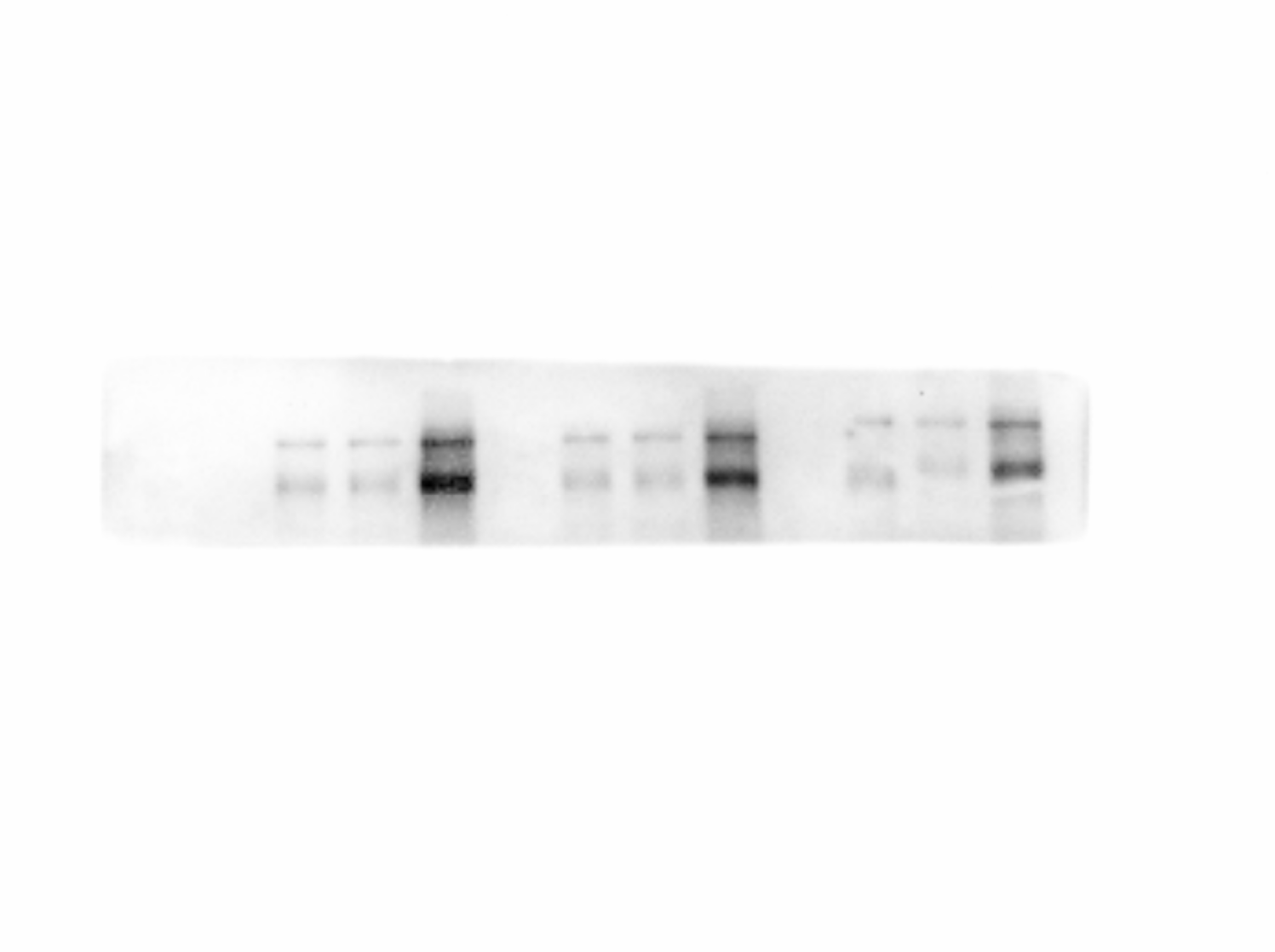


CD63


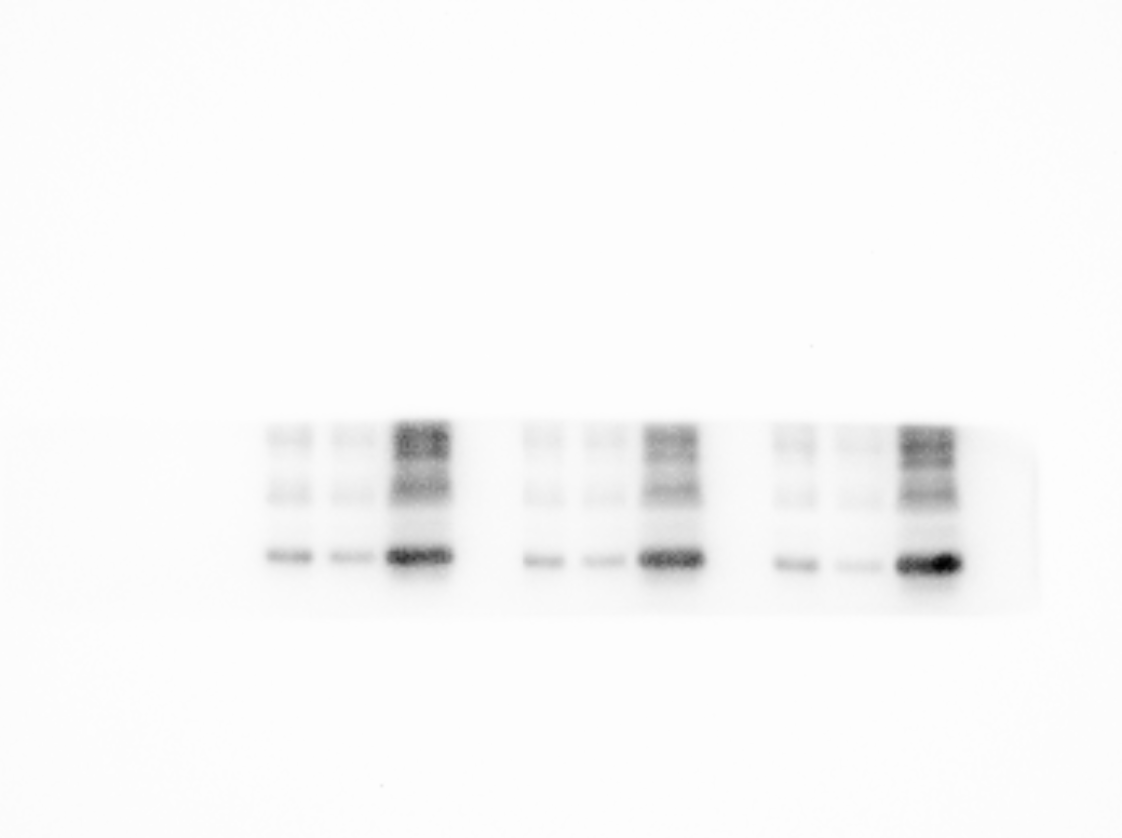


ALB


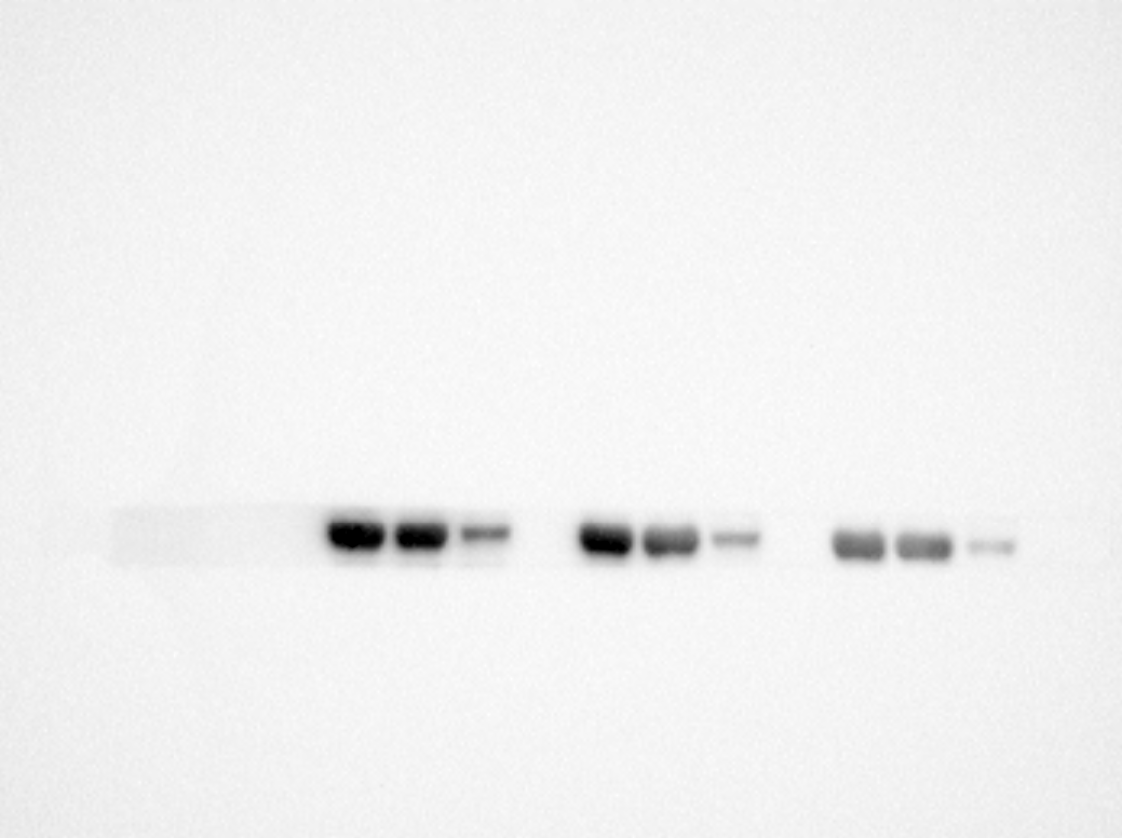


Figure 6

N-Cadherin


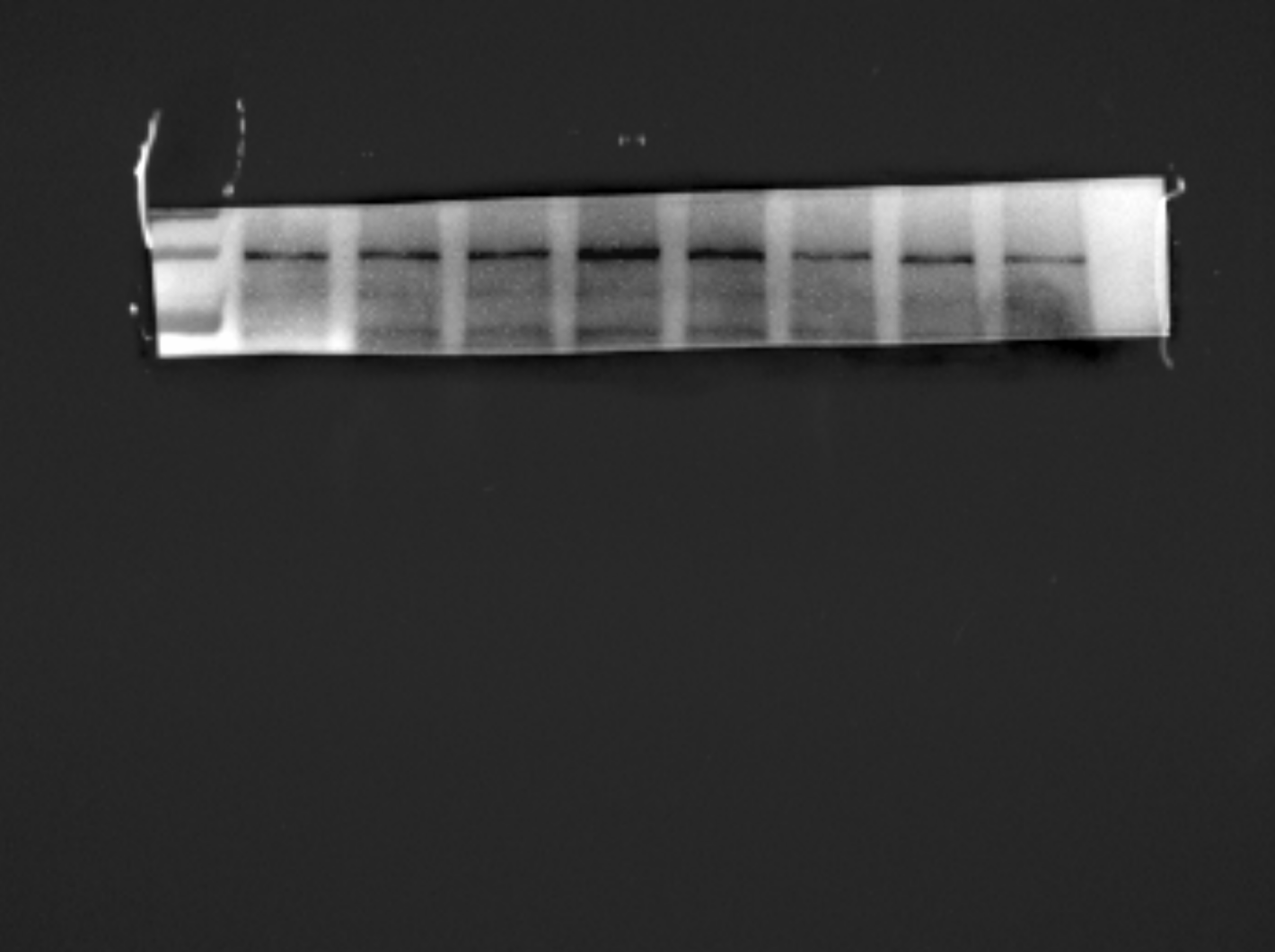


GSK3β


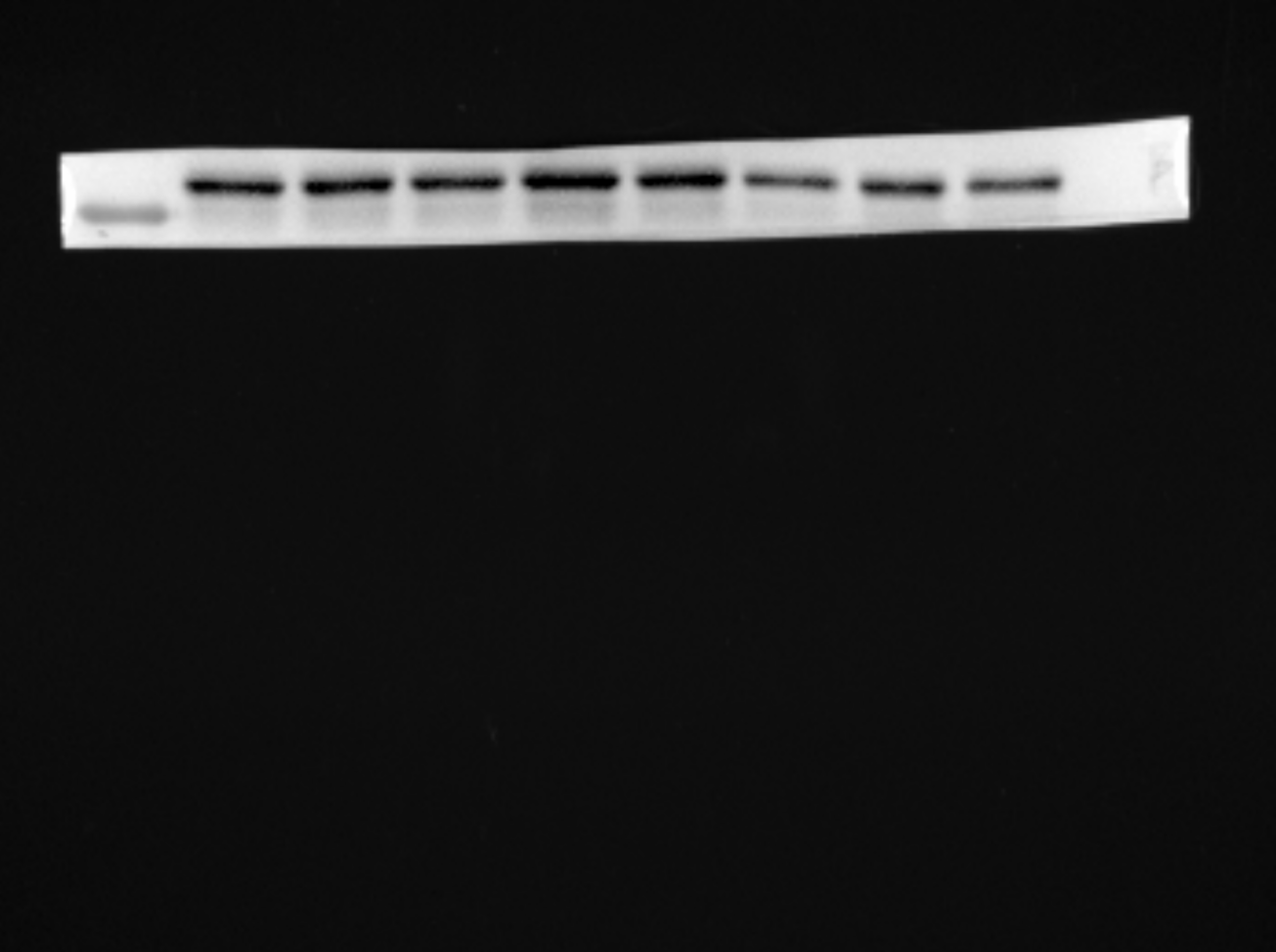


Slug


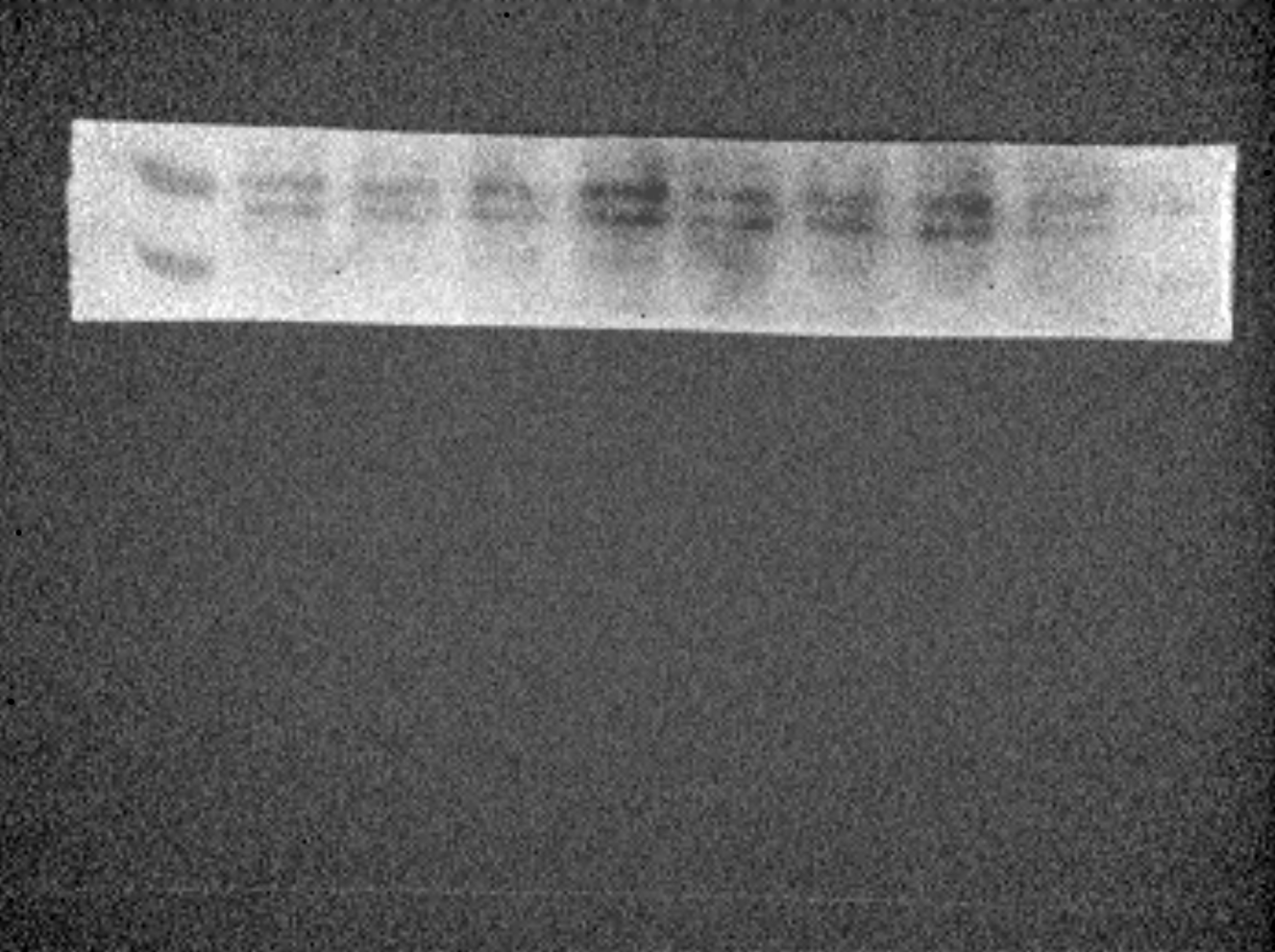


MMP-9


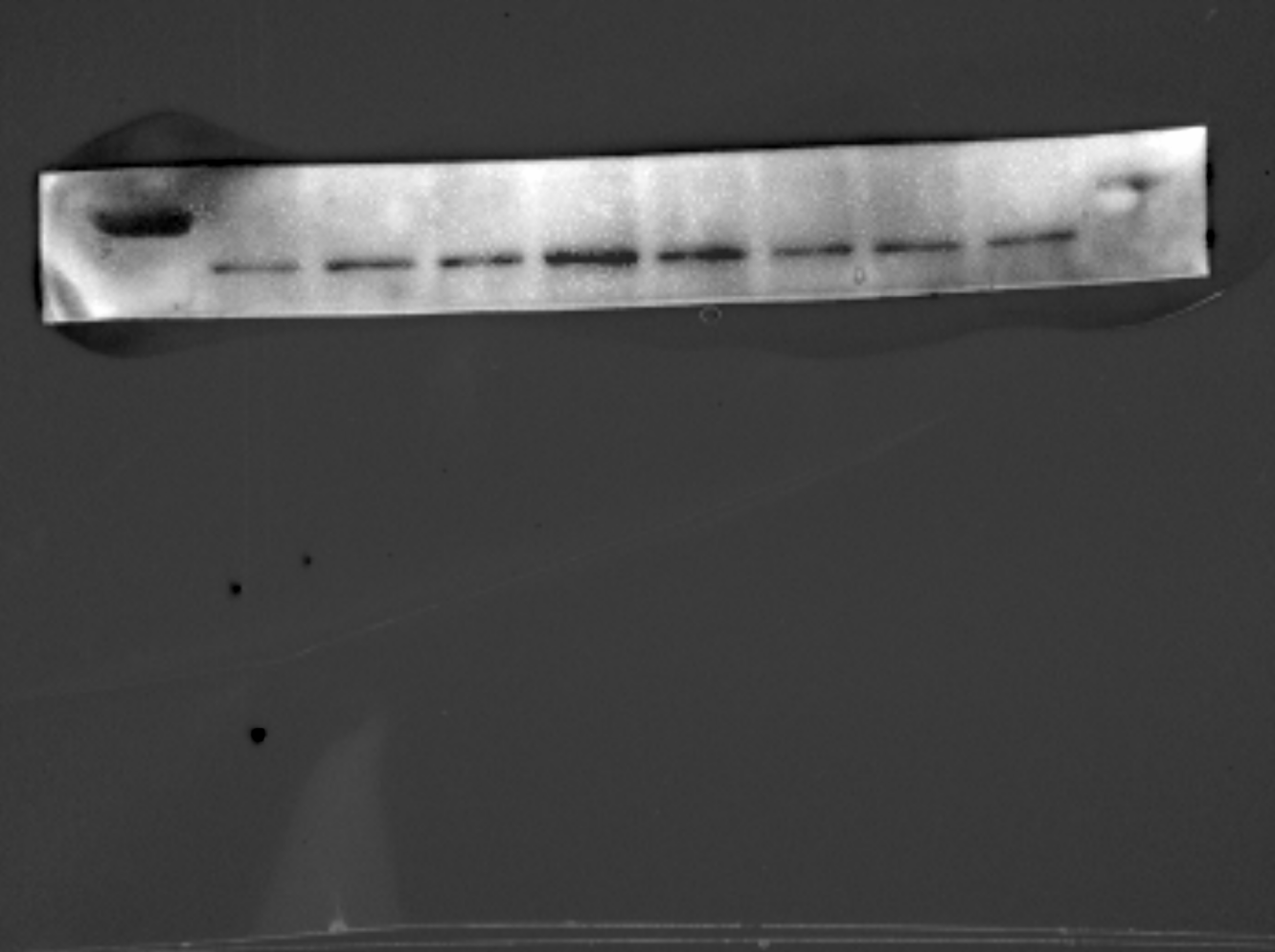


NK-κB


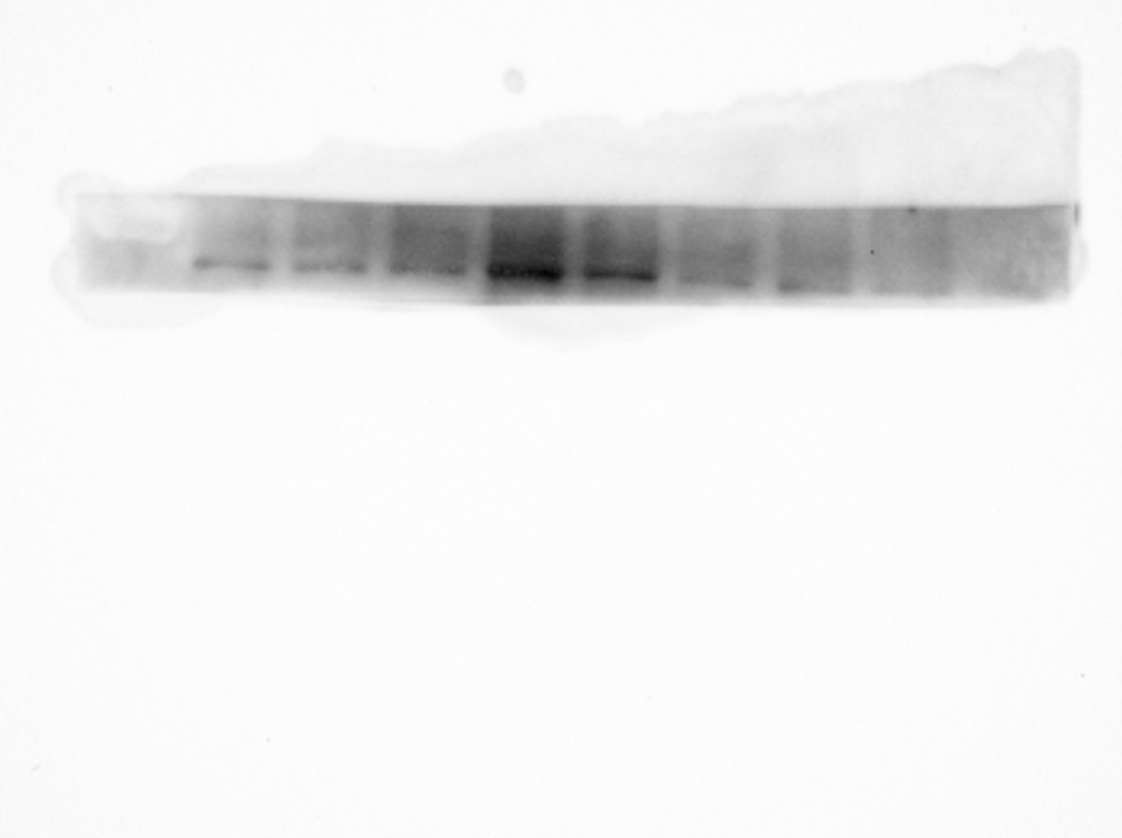


β-actin


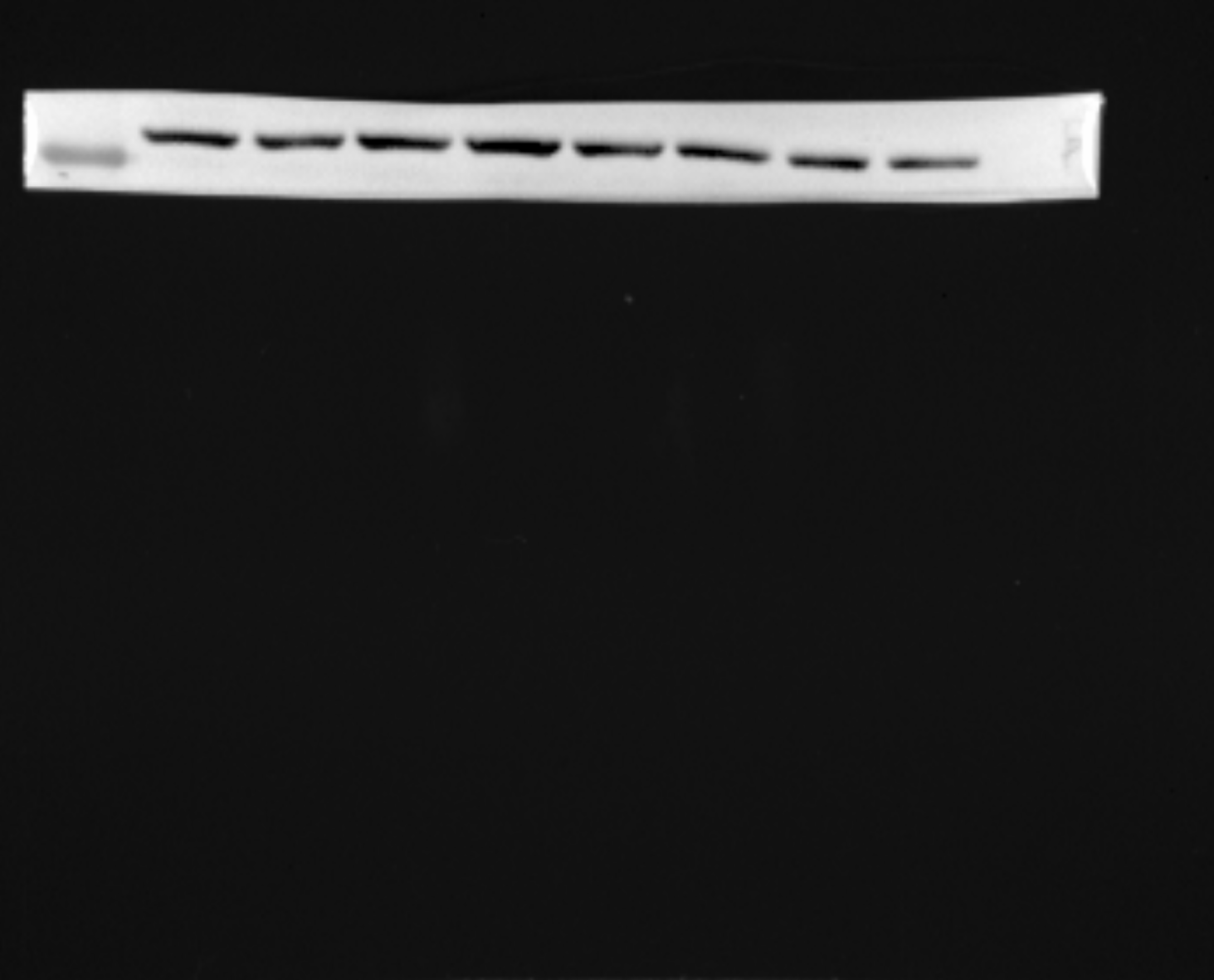

Supplement: Supplementary file 1 [file DataSheet_1.docx]

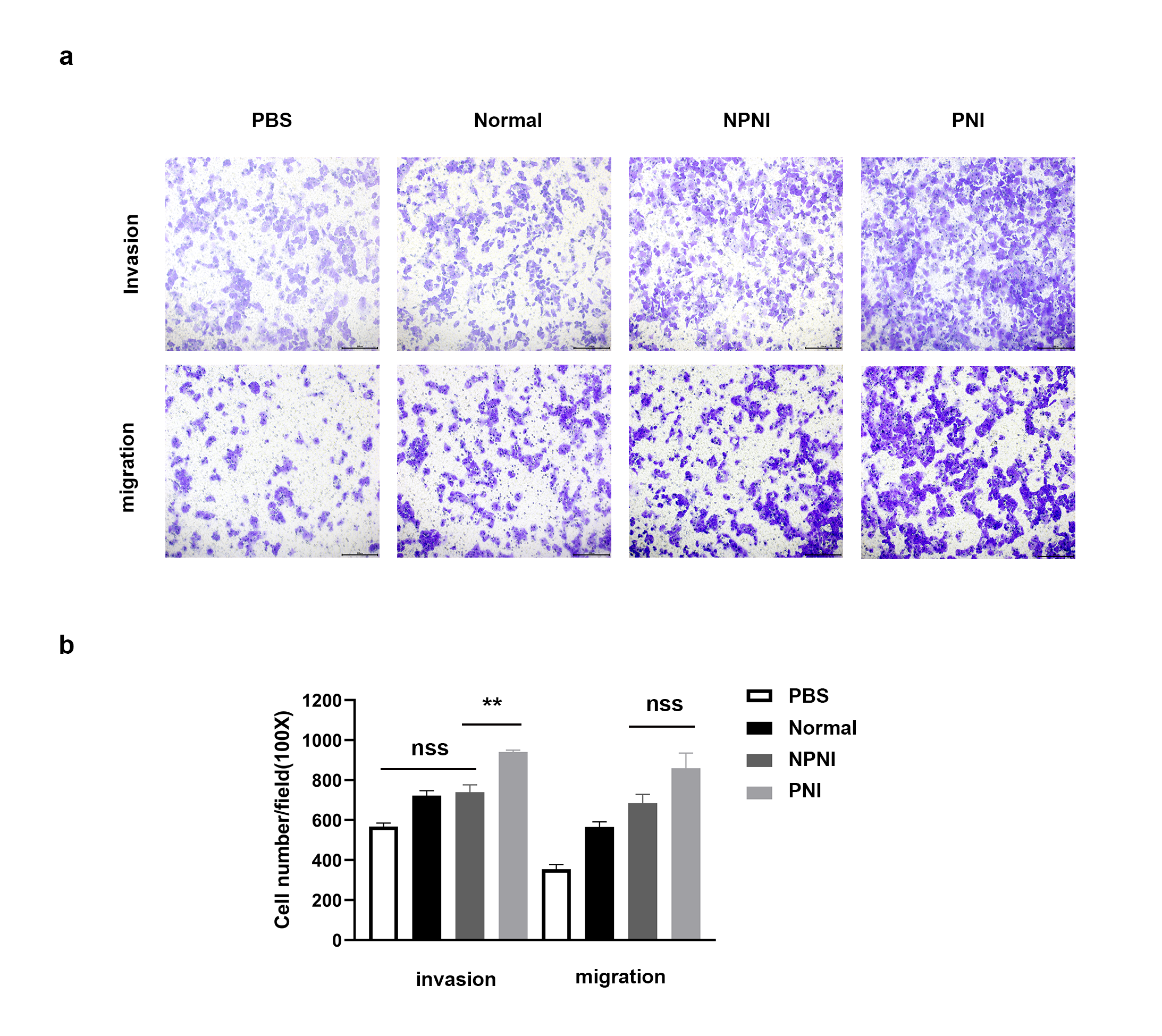

Supplement: Supplementary file 2 [file Image_1.png]

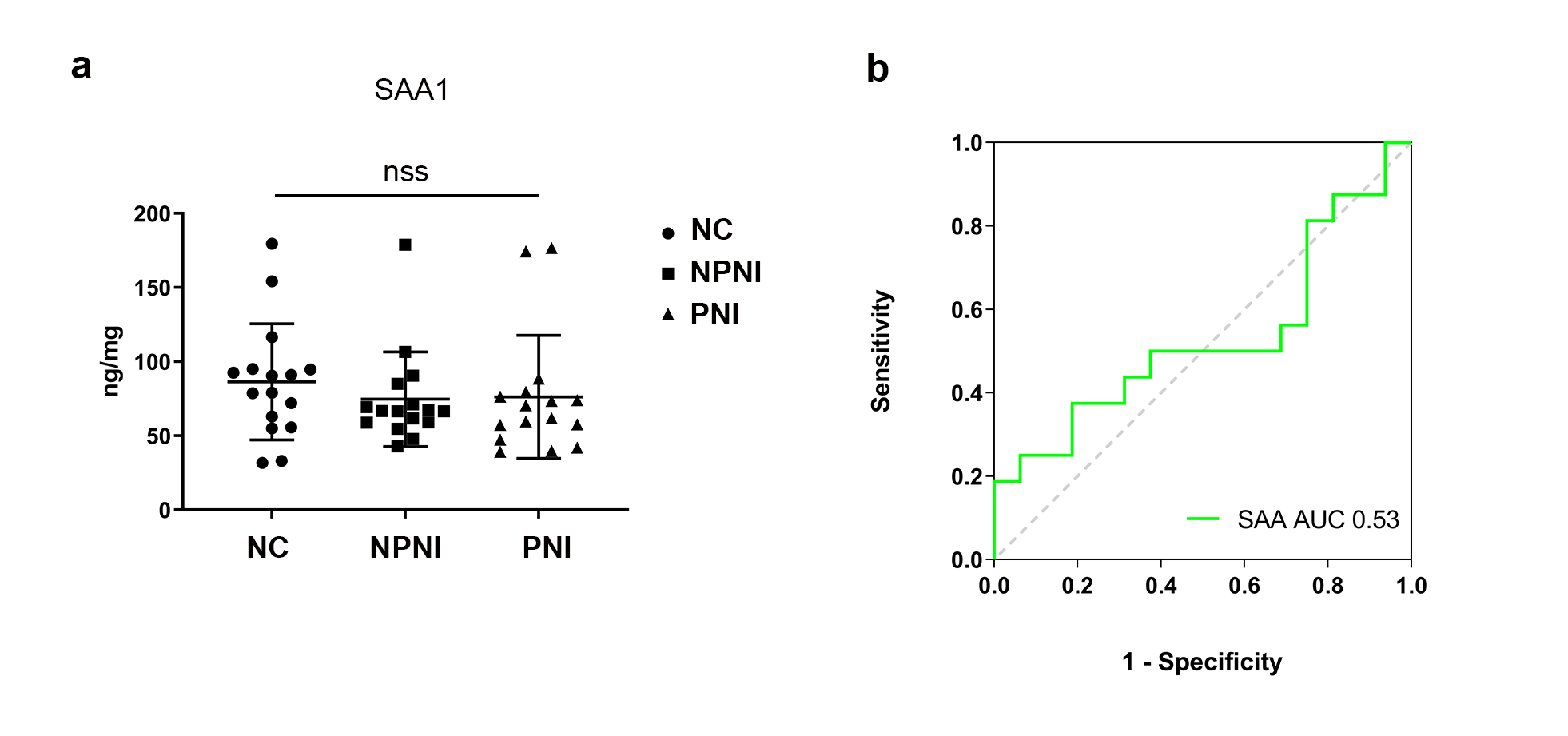

Supplement: Supplementary file 3 [file Image_2.png]
